# A full utilization of rice husk to evaluate phytochemical bioactivities and prepare cellulose nanocrystals

**DOI:** 10.1038/s41598-018-27635-3

**Published:** 2018-07-11

**Authors:** Yue Gao, Xinbo Guo, Yu Liu, Zhiqiang Fang, Mingwei Zhang, Ruifen Zhang, Lijun You, Tong Li, Rui Hai Liu

**Affiliations:** 1 School of Food Science and Engineering, South China University of Technology, Guangzhou, 510640 China; 2 School of Pulp and Paper Engineering, South China University of Technology, Guangzhou, 510640 China; 3 Sericultural & Agri-Food Research Institute Guangdong Academy of Agricultural Sciences/Key Laboratory of Functional Foods, Ministry of Agriculture/Guangdong Key Laboratory of Agricultural Products Processing, Guangzhou, 510610 PR China; 4 Department of Food Science, Stocking Hall, Cornell University, Ithaca, New York 14853 USA

## Abstract

Rice husks (RHs) as an agro-waste generated from rice production, while its application is limited. This study was designed to introduce a full utilization of rice husks, which extracted the phytochemical at first and then produced cellulose nanocrystals (CNCs) as the use of the residue. Furthermore, the phytochemicals extracted from rice husk was identified and its biological activity, including antioxidant activity, cellular antioxidant activity (CAA) and antiproliferative activity, had been detected as well. Results showed the bound fraction of rice husk had higher antioxidant than common fruit and grain. Free fraction of rice husk deserved to have further analysis in antiproliferative activity due to its low cytotoxicity. The CNCs produced by residue was using delignification process and acid hydrolysis treatments. The chemical composition of the residue obtained after phytochemical extraction was determined. CNCs morphological investigation was performed using an optical microscope and atomic force microscopy (AFM). Our strategy is to achieve a comprehensive utilization of rice husks with both economy and environment benefits.

## Introduction

The utilization of waste material and byproduct plays a vital part in solving economic issues at present. Rice husk is the byproduct of rice milling and the major agro-waste from rice production. World production of rice annually was 741 million tons, and approximately 148 million tons of husks were generated, corresponding to 20% of grain weight^1^. However, due to their tough structure and huge bulk, rice husk has limited applications and was treated by burying underground or open field burning. Thus, it is of great benefit to both the economy and environment to effectively convert rice husk into valuable products.

Recently researches regarding obtaining higher values from RHs have been investigated, such as deriving bioactive phytochemicals due to its protection against oxidative damage^2,3^. The strong link between antioxidant activity and phytochemical especially phenolics had been demonstrated by numerous reports, which indicate that phenolic compounds could be the dominant factor of antioxidant capacity^4^. Furthermore, phenolic compounds were found to inhibit the growth of tumor cell lines from human cancers^5^. Several literatures had been reported the *in vitro* antioxidant activity and phenolic compounds of rice husk^2,6^. However, none of these researches reported the cellular antioxidant activity of rice husk. Moreover, the antiproliferative activity of phytochemical extracted from rice husk was limited.

In addition, agricultural solid residues with high cellulose content have been proposed to use in cellulose and nanocellulose production^7,8^. Studies have shown that rice husk contains about 35–40% cellulose, 15–20% hemicellulose, and 20–25% lignin^9^. Hence, another way to take advantage of rice husk includes the utilization of the cellulose section. Johar *et al*.^10^ had successfully produced cellulose fibers and nanocrystals from rice husk using an acid hydrolysis treatment. CNCs has attracted a great deal of attention for its wide application, including reinforced nanocomposites, optically transparent functional materials^11,12^. Moreover, their use in films, membranes, catalyst support materials, and functionalized drug carriers has being explored recently^13^. In the study of Wijaya *et al*.^14^, CNC was extracted from passionfruit peels and used as tetracycline antibiotic drug carrier. Therefore, the use of rice husk as the primary source for producing cellulose nanocrystals is promising.

Thus, the objective of this study was to introduce a comprehensive approach to utilize rice husk by deriving phytochemicals primarily and gaining CNCs from residue. Moreover, phenolic compounds, antioxidant and antiproliferative activities were evaluated and morphological investigation of the CNCs was conducted. Through this method, phytochemical extracted from rice husk can be a potential ingredient in antioxidants or anticancer products. And the CNC made from the residue can be applied to other areas that generate economic value such as reinforcement materials and drug carriers.

## Results and Discussion

### Phenolic and Flavonoid Contents of Rice Husk

The contents of phenolic and flavonoid of rice husk were presented in Table 1. The bound fraction was 91.95 and 76.30% contribution to the total phenolics and flavonoids, respectively, which demonstrated that most of the phytochemicals were existed in bound fraction both in phenolic and flavonoid groups. Consistent with our result, Wanyo *et al*.^2^ claimed that bound phenolic contents of rice husk extracted by acetone accounted for 73.3 and 80.0% contributes to total phenolic and flavonoid contents, respectively. In addition, the content of phenolics had little difference compared to the content of flavonoids in free fraction. The total phenolic and flavonoid contents of rice husk were 14.90 ± 0.7 mg GAE/g and 3.08 ± 0.17 mg CE/g, which were consistent with results reported by Seungcheol *et al*.^15^. It is known that corn has a higher total phenolic content in whole grain and its total phenolic content was about 2.65 mg GAE/g^16^, which accounted for about 20% of that in rice husk. The same phenomenon was observed between some kinds of common fruit^17^, vegetable^18^ and rice husk, which strongly indicated the potential biological activity in rice husk extracts.

**Table 1:** The content and phenolic profile of free, bound and total phenolics and flavonoids of rice husk.

	free	bound	total
total phenolics (mg GAE/g)	1.20 ± 0.06 (8.05%)^a^	13.70 ± 0.67 (91.95%)	14.90 ± 0.70
total flavonoids (mg CE/g)	0.73 ± 0.07 (23.70%)	2.35 ± 0.12 (76.30%)	3.08 ± 0.17
*p*-hydroxybenzoic acid (mg/100 g)	1.39 ± 0.06	11.16 ± 0.94	12.55 ± 0.93
caffeic acid (mg/100 g)	0.38 ± 0.02	3.30 ± 0.13	3.68 ± 0.14
*p*-coumaric acid (mg/100 g)	1.01 ± 0.02	264.4 ± 2.4	265.4 ± 2.4
ferulic acid (mg/100 g)	1.03 ± 0.07	32.61 ± 1.02	33.64 ± 1.01

Data are expressed as mean ± standard deviation of triplicate samples.

^a^Values in parentheses indicate percentage contribution of this fraction to the corresponding total fraction.

### Phytochemical Profiles of Rice Husk

**Figure 1:**
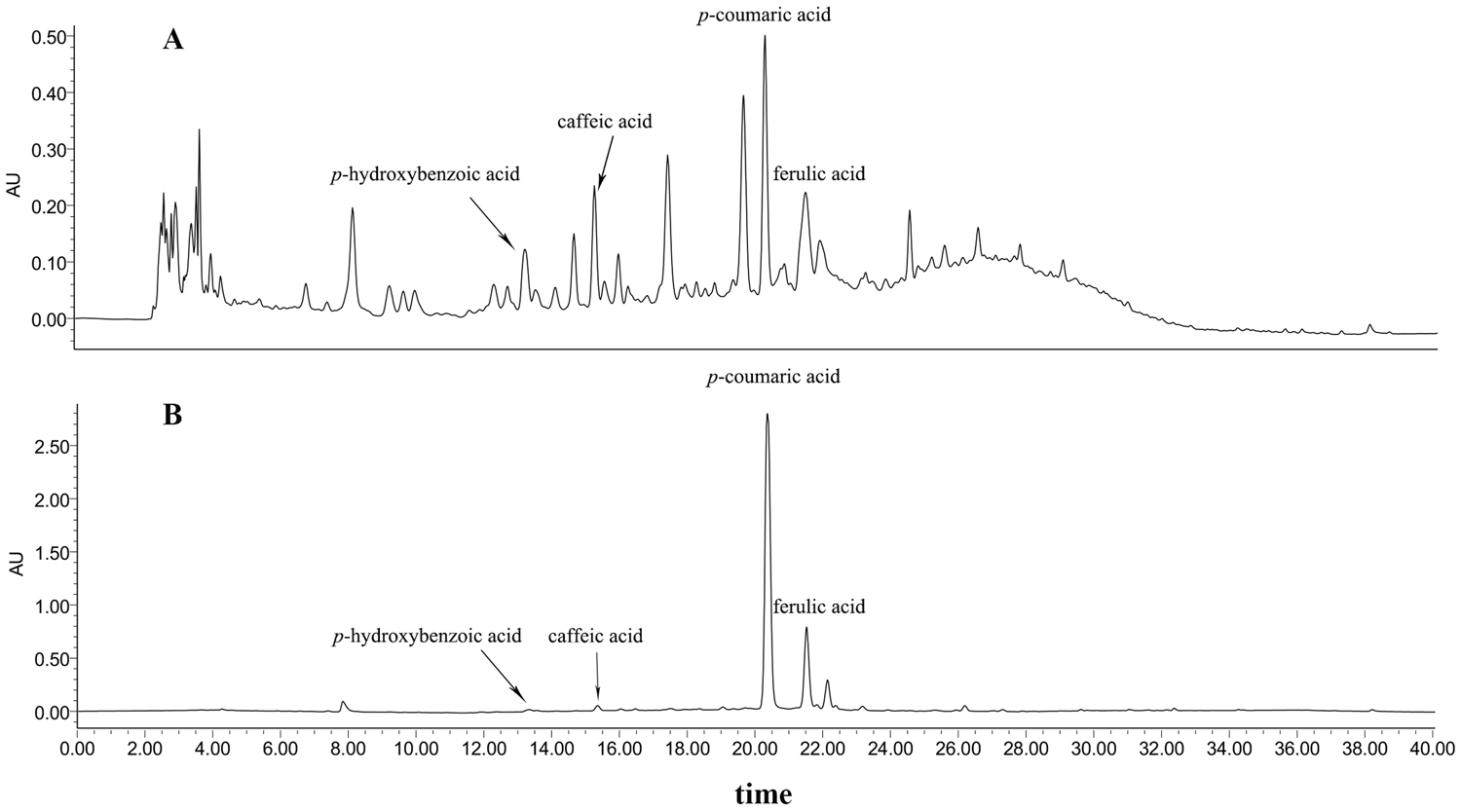
HPLC chromatograms of free (**A**) and bound (**B**) phenolic compounds in rice husk. Flow rate: 1.0 mL/min; Detection is at 280 nm.

The HPLC chromatogram of free and bound phenolic acids of rice husk was shown in Fig. 1. Four phenolic acids, *p*-hydroxybenzoic acid, caffeic acid, *p*-coumaric acid and ferulic acid, were identified and quantified and their contents were summarized in Table 1. As shown in Fig. 1A, a wide variety of the phytochemical components was observed in free fraction of rice husk, while there was little difference in their content. However, an opposite tendency was displayed in bound fraction in Fig. 1B. *P*-coumaric acid and ferulic acid were the major phenolic acids in bound fraction and the contents were 265.4 ± 2.4 and 33.64 ± 1.01 mg/100 g, respectively. This data was significantly higher than those in brown rice, millet, and other cereals^19–21^. Previous reported showed the antiperoxidative potential of *p*-coumaric acid against adjuvant-induced arthritis in rats^22^. Janicke *et al*. also reported ferulic acid and *p*-coumaric acid could produce antiproliferative effects by acting on the cell cycle of Caco-2 cells^23^. Our data demonstrated the high content of *p*-coumaric acid and ferulic acid in rice husk extract, therefore, the antioxidant and antiproliferative activities of phytochemical extracted from rice husk were evaluated below.

### *In vitro* and Cellular Antioxidant Activity of Rice Husk

**Figure 2:**
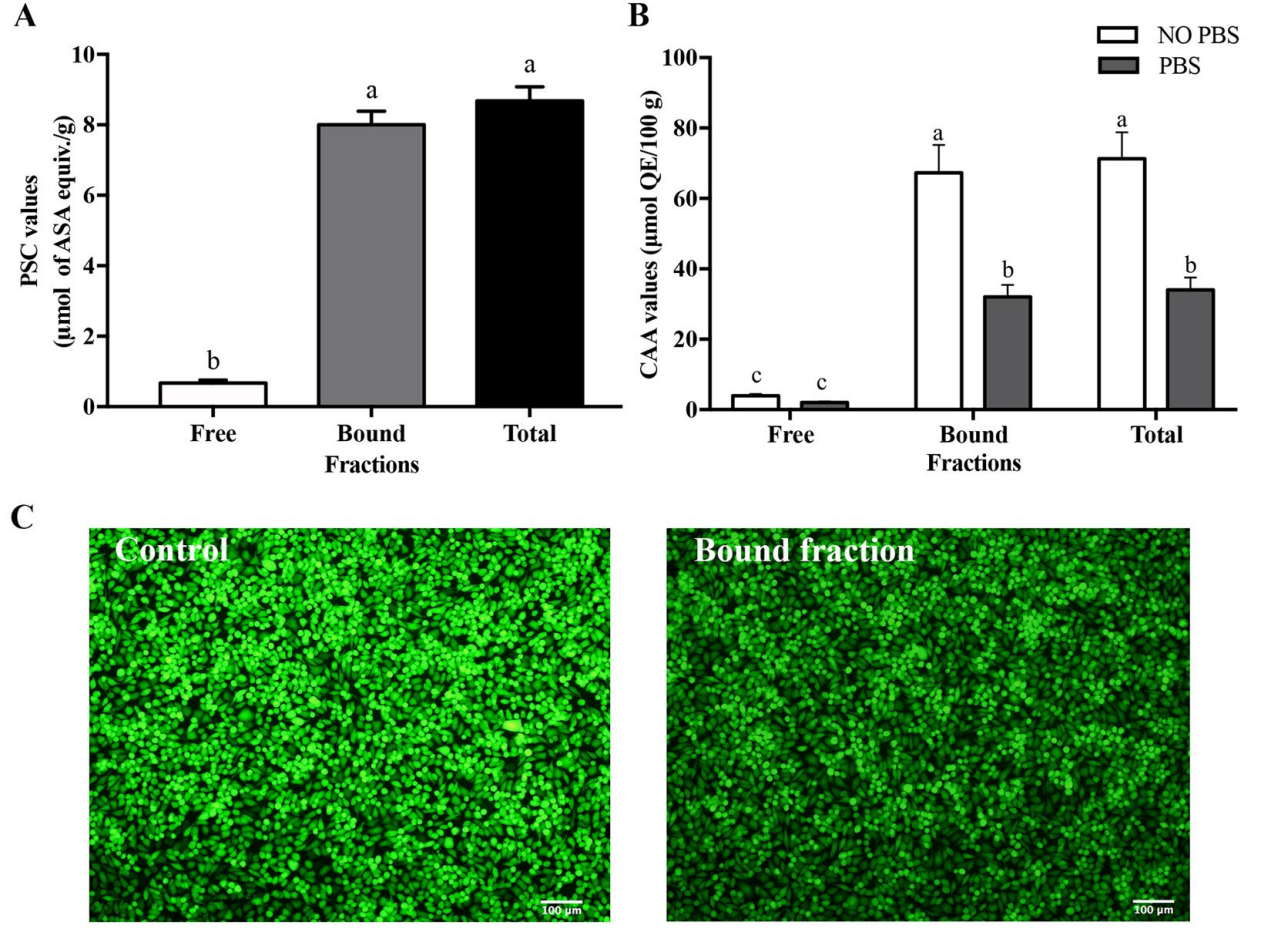
Antioxidant activity of the free, bound and total fraction of rice husk. (**A**) Antioxidant activity of rice husk expressed as PSC values (mean ± SD, n = 3). Bars with no letters in common are significantly different (*p* < 0.05); (**B**) Cellular antioxidant activity of rice husk expressed as CAA values (mean ± SD, n = 3). Bars with no letters in common are significantly different (*p* < 0.05); (**C**) The fluorescence picture of control and bound fraction of rice husk taken by fluorescence microscopy (Olympus IX83 Inverted Microscope).

PSC and CAA assays were used here to quantify the *in vitro* antioxidant and cellular antioxidant activity of rice husk extract, respectively. On the whole, the PSC value (Fig. 2A) of the bound fraction had a dominant effect in samples, accounting for approximately 90% contribution to the total PSC values, which was in accordance with the content of phenolic. The high consistence between *in vitro* antioxidant activity and total phenolic contents were similar to previous study reported by Zhang *et al*.^24^ Furthermore, the total PSC value of rice husk extracts was 8.68 ± 0.40 μmol ASA equiv./g, which was about 3.98 times higher than that of bound fraction of whole rice^25^, respectively. Our results indicated that the antioxidant of rice husk extracts, especially bound fraction, had higher antioxidant activity.

The CAA assay is a more biologically relevant method because it simulates some of the cellular processes including cell uptake, metabolism, and distribution to predict the antioxidant behavior in biological systems^26^. PBS wash protocol and no PBS wash protocol were used to detect the degree of uptake and membrane association of samples. The CAA values (Fig. 2B) for bound fractions were 67.27 ± 7.94 and 32.00 ± 3.43 μmol QE/100 g in no PBS wash and PBS wash protocols, respectively, which were significantly higher than corresponding free fractions (3.99 ± 0.45 and 2.00 ± 0.36 μmol QE/100 g, respectively). The total CAA value of rice husk extracts was approximately 1.48 and 3.70 times higher than that of cranberry and red grape in no PBS protocol. Also, approximately 2.27 and 3.09 times higher than above fruit had been found in PBS protocol^26^. The fluorescence picture (Fig. 2C) taken by fluorescence microscopy (Olympus IX83 Inverted Microscope) could observe the antioxidant activity of bound fraction more directly. Bound fraction of rice husk could prevent oxidation of DCFH and reduced the formation of the fluorescent DCF, in that produce a darker field of view than control. The high cellular antioxidant activity of rice husk extracts demonstrated the potentials of rice husk, especially the bound fraction of rice husk, as natural sources of antioxidants with health benefits. Butsat and Siriamornpun also reported that rice husk can be regarded as valuable sources of bioactive components due to its high antioxidant properties^3^.

### Antiproliferative Activity of Rice Husk

**Figure 3:**
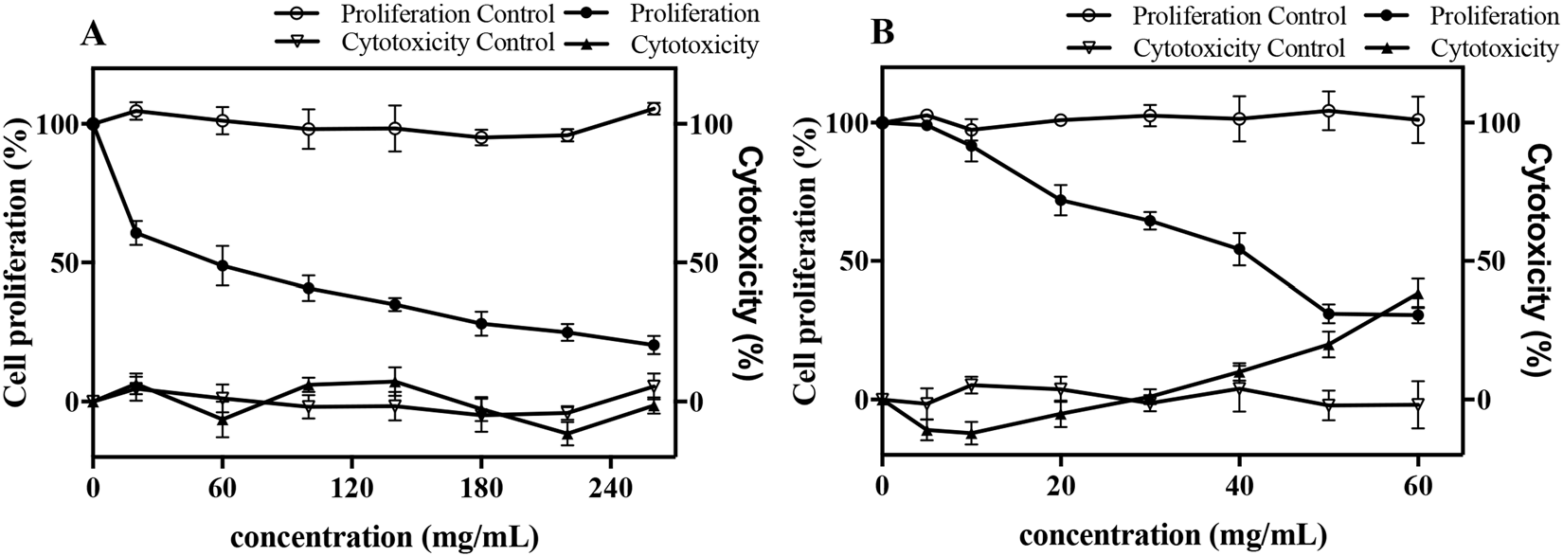
Antiproliferative activity and cytotoxicity of free (**A**) and bound (**B**) fraction of rice husk against HepG2 human liver cancer cells (means ± SD, n = 3).

The antiproliferative activities and cytotoxicity were evaluated by HepG2 cells and shown in Fig. 3. On the whole, the inhibitions of HepG2 cells proliferation by the free (Fig. 3A) and bound (Fig. 3B) fractions were shown in a dose-dependent manner. At the concentration of 260 mg/mL, free fraction of rice husk showed no cytotoxicity and its cell proliferation was inhibited by 80%. At the concentration of 40 mg/ml, bound fraction of rice husk did not exhibited cytotoxicity, but inhibited cell proliferation by 54% specificly. Final values of antiproliferative activity and cytotoxicity were presented as the median inhibition dose (IC_50_) and the median cytotoxicity dose (CC_50_). The IC50 value of free fraction was 46.90 ± 2.79 mg/mL, while bound fraction had a higher antiproliferative activity at IC_50_ 37.58 ± 4.40 mg/mL and at CC_50_ 67.86 ± 1.62 mg/mL. It was observed that concentration at IC_50_ of free fraction showed no cytotoxicity, which indicated that the inhibitory effect of free fraction was not due to cytotoxicity but to the antiproliferative effect. Previous researches indicated *p*-coumaric can inhibit MCF-7 cells proliferation with an EC50 value at 1856.90 ± 70.25 μM^27^, and ferulic acid at 10 μM concentration can inhibit 21.09% growth of HepG2 cells^28^. Results indicated that the antiproliferative activitiy of the free fraction of rice husk deserved to have further study due to its low cytotoxicity. The antiproliferative activity of free and bound fraction revealed less consistence to phenolic content and antioxidant activity, which was in accord with the report of Nzaramba *et al*.^29^, who found no significant correlations between antiproliferative activity and antioxidant activity, phenolics in both HT-29 and LNCaP prostate cancer cell lines. This may be because of different effects, such as additive, synergistic and/or antagonistic effects among component presented in rice husk extracts. Our result demonstrated that the antiproliferative activities could not be explained only by their phenolic compound contents; Besides, the interaction among components presented in extracts should be considered^30^.

### Chemical composition and morphological investigation of the CNCs

**Figure 4:**
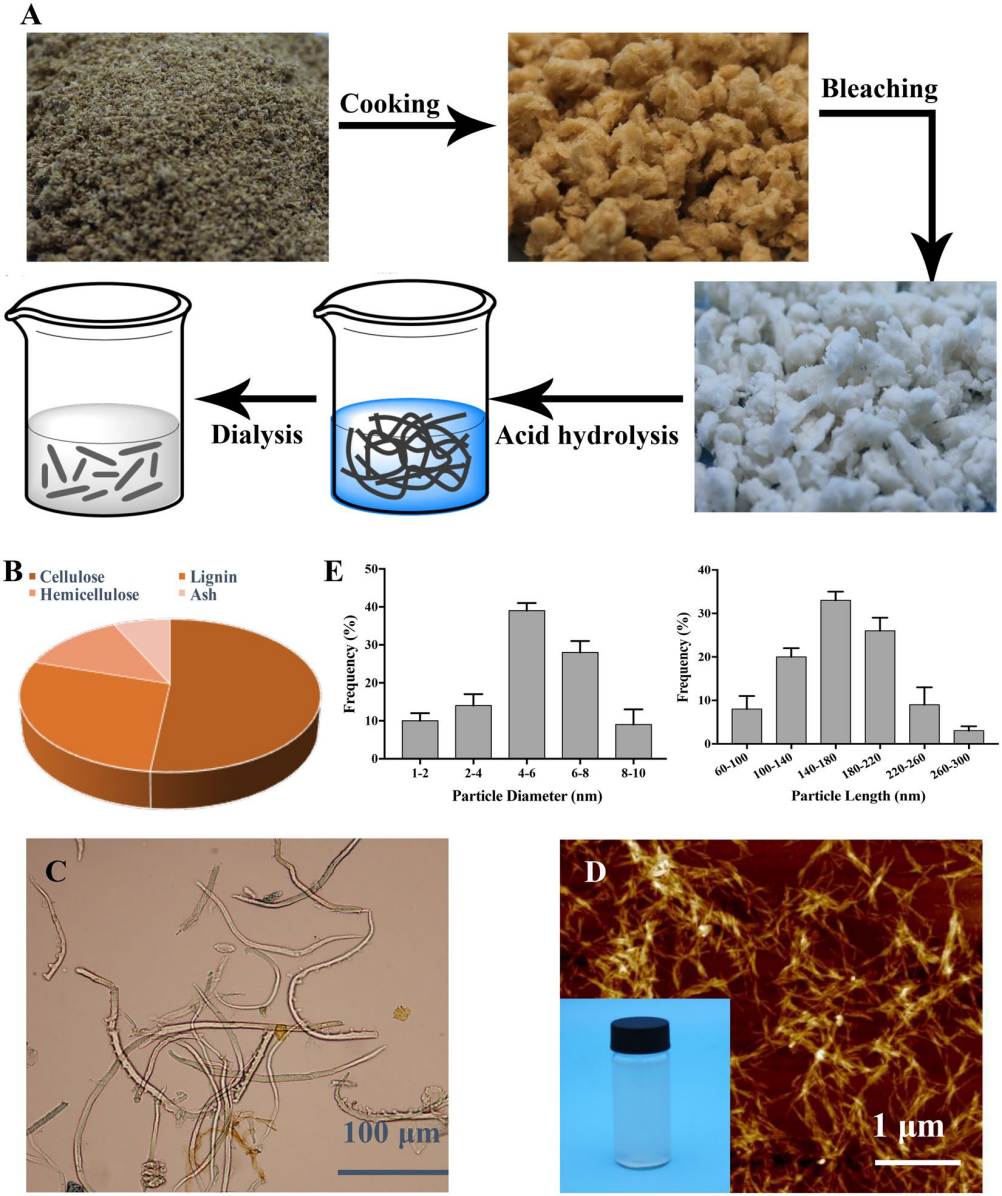
Chemical composition and morphological investigation of the CNCs. (**A**) Preparation of CNCs from the residue of rice husk; (**B**) Chemical composition of the residue; (**C**) Micrographs of the fibers after bleached; (**D**) Height mode AFM images of CNCs and picture of the real CNCs; (**E**) The frequency of particles diameter (nm) and length (nm) of CNCs.

The chemical composition of rice husk was given in Supplementary file. The content of cellulose, hemicellulose, lignin, and ash were detected after phytochemical extracted from rice husk, as shown in Fig. 4B. The cellulose content of residue was above 50%, which suggested the promising production of CNCs. The procedure of CNCs production from residue was observed in Fig. 4A and details of cooking, bleaching, acid hydrolysis and dialysis were given in Materials and methods. Moreover, an optical microscope was used to observe the width and length of fibers obtained after bleaching treatment (Fig. 4C). Results showed the widths of bleached fiber were 5–15 μm and the corresponding lengths were 0.1–1 mm. The CNCs produced from residues was verified by AFM image and presented in Fig. 4D. The reason of agglomerated aspect of CNCs observed in the figure may be due to their high specific area and the strong hydrogen bonds between crystallites^31^. Furthermore, more than a hundred particles were chosen randomly and the diameter and length of these were measured, which was shown in Fig. 4E. A broad polydispersity, with a length ranging from 60 to 300 nm and a diameter between 1 and 10 nm, was observed. Diameter at 4–8 nm and length at 100–220 nm accounted for approximately 70% of the total. The application of CNCs obtained from rice husk was broad. Ooi *et al*. produced CNC-gelatin hydrogels to act as drug carriers, which could be used in controlled drug delivery systems^32^. Tang *et al*. also found the potential of polyrhodanine coated cellulose nanocrystals to be antimicrobial agent^33^.

**Figure 5:**
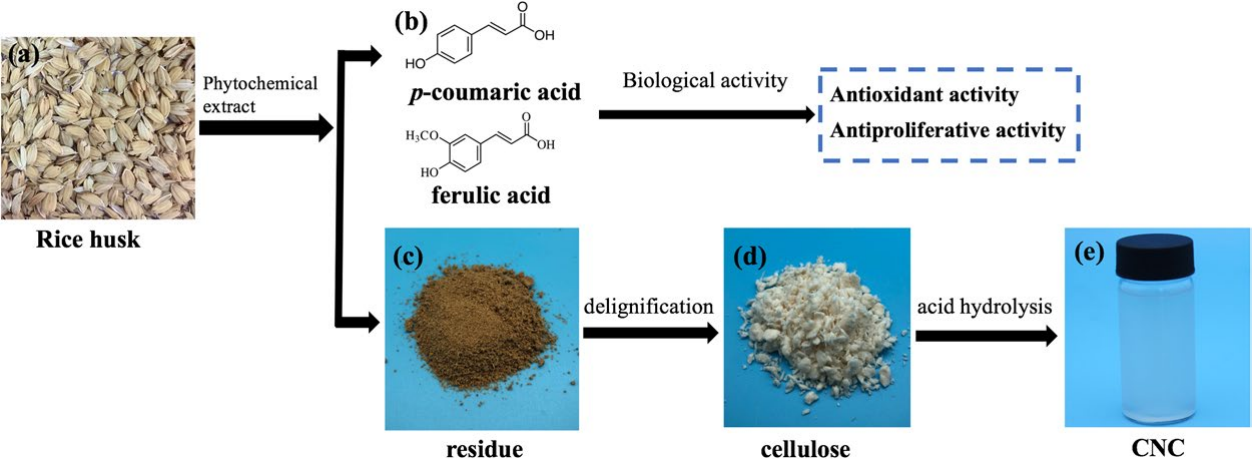
A flowchart of rice husk full utilization. (**a**) the photo of rice husk; (**b**) the main phenolic compounds in rice husk; (**c**) the picture of residue after phytochemical extraction; (**d**) the cellulose gained after delignification; (**e**) the CNC prepared by acid hydrolysis from cellulose of rice husk.

In conclusion, this work presented a fine example of the full application of rice husk, which is expected to promote effective utilization of ago-waste in the near future (Fig. 5). Our results reveal that the potentials of phenolic compounds extracted from rice husk as natural sources of antioxidants in bound and free fractions. Cellulose nanocrystals were successfully extracted from the residue after phytochemical extraction using an acid hydrolysis treatment, which was confirmed by chemical composition determination, morphological investigation. Rice husk as a wasted product can be used in the manufacture of nutritional supplements, the antimicrobial agent, food-packaging and other possible applications.

## Materials and Methods

### Materials and Reagents

Rice husk was provided by the Rice Research Institute of Guangdong Academy of Agricultural Science, China. Folin-Ciocalteu reagent, fluorescein disodium salt, 2,2′-azobis (2-amidinopropane) dihydrochloride (ABAP), 2′,7′-Dichlorofluorescin diacetate (DCFH-DA), (+)-catechin, gallic acid, and chromatographic grade of *p*-coumaric acid, caffeic acid, *p*–coumaric acid, ferulic acid were purchased from Sigma-Aldrich Ltd. (St. Louis, MO, USA). Chromatographic grade of acetonitrile used for HPLC analysis was obtained from Anpel Ltd. (Shanghai, China). Human liver cancer cell line HepG2 (ATCC HB-8065) were purchased from ATCC company (Manassas, VA, USA). WME medium, fetal bovine serum (FBS), Hank’s balanced salt solution (HBSS), Trypsin-EDTA solution and other cell culture reagents were purchased from Gibco Life Technologies Co. (Grand Island, NY, USA).

### Extraction of Free and Bound Phenolic Compounds

Phenolics were extracted according to previously reported with a slight modification^34^. Briefly, the free phenolics in rice husk were extracted by chilled 80% acetone and then reconstituted using 70% methanol. The bound phenolics were digested and neutralized primarily, and then extracted five times with ethyl acetate. The ethyl acetate fractions reconstituted using 70% methanol. Both extractions and the remaining residue were stored at −40 °C for further use.

### Determination of Phenolics and Flavonoids

The phenolic content was measured by Folin-Ciocalteu method as reported previously^35^ and gallic acid was used as the standard. The flavonoids were determined using the sodium borohydride/chloranil assay (SBC)^36^, using catechin as the standard. Data is expressed as milligram gallic acid equivalent per gram (mg GAE/g) and milligram catechin equivalent per gram (mg CE/g) of rice husk in triplicate, respectively.

### Analysis of Phytochemical Composition

The identification of phytochemical was performed according to the method reported previously^37^ with slight modifications. The samples were analyzed by an HPLC system (Waters Co., USA), which was equipped with a 4.6 × 250 mm, 5 μm Sunfire C_18_ reversed phase column (Waters, USA). The mobile phase consisted of purified water with 0.1% trifluoroacetic acid (solvent A) and acetonitrile (solvent B) at a flow rate of 1.0 mL/ min using gradient elution as follows: 0–5 min 10% B, 5–20 min 25% B, 20–25 min 35% B, 25–40 min 90% B, 40–50 min 10% B, 50–60 min 10% B at 280 nm. Phenolic compounds in rice husk were identified by comparing the retention time and detected using an external standard method. Measured values were expressed as milligrams per 100 g of rice husk (mg/100 g).

### Quantification of *in vitro* Antioxidant Activity

The *in vitro* antioxidant activity was evaluated by the peroxyl radical scavenging capacity (PSC) assays as described previously^25^. Ascorbic acid (ASA) was used as the standard. In brief, diluted samples and standard solutions were mixed with equivalent volume DCFH–DA and followed by ABAP. The reaction was performed at 485 nm excitation and 538 nm emission by the multi-mode microplate reader (Molecular Devices, Orleans Drive Sunnyvale, CA, USA). Results were calculated as micromoles of ascorbic acid equivalent per gram (μmol ASA equiv./g) and the data were reported as mean ± SD (n = 3).

### Measurement of Cellular Antioxidant Activity

Human liver cancer cells HepG2 were cultured in WME medium supplemented with 2 mM L-glutamine, 10 mM Hepes, 5% fetal bovine serum, 5 μg/mL insulin, 50 units/mL penicillin, 0.05 μg/mL hydrocortisone, 50 μg/mL streptomycin and 100 μg/mL gentamycin at 37 °C in a humidified atmosphere of 5% CO_2_.

The CAA assay was conducted as described previously^26^. Quercetin was used as the standard. In brief, HepG2 cells were incubated at a density of 6.0 × 10^4^ cells/well for 24 h and followed by medium containing samples which were under the cytotoxicity dose plus 25 μM DCFH-DA. ABAP was added after incubated for 1 h and the fluorescence intensity was monitored at 485 nm excitation and 535 nm emission every 5 min for 1 h (Molecular Devices). The CAA value was presented as micromoles of quercetin equivalent per 100 g of rice husk (μmol QE/100 g).

### Cytotoxicity and Antiproliferative Activity Assays

The antiproliferative activity was measured in HepG2 cells by using a methylene blue assay that has been reported previously^38^. Briefly, cells were seeded at a concentration of 2.5 × 10^4^ cells/well. For cytotoxicity test, cells were plated at the concentration of 4 × 10^4^ cells/well. After incubated, a series concentration of diluted extracts in growth medium was added into each well and then stained for viable number counting. Absorbance was measured at 570 nm on Multi-Mode Microplate Reader (Molecular Devices). Each sample was measured at least three times. The cytotoxicity and antiproliferative activity of phytochemicals were assessed by half maximal cytotoxicity concentration (CC_50_) and median inhibition dose (IC_50_), respectively, which were expressed as mg/ml (mean ± SD, n = 3).

### Chemical Composition of The Residue

The chemical composition of the residue was determined according to the Technical Association of Pulp and Paper Industry (TAPPI). T203 OS-74^39^ was used to measure the content of cellulose and hemicellulose and the lignin content was obtained according to T222 OS-83^40^. The ash content was determined after pyrolysis of the dry sample in a furnace at 525 °C for 6 h, following the standard procedure of T211 om-02^41^.

### Preparation of Cellulose Nanocrystals from Residue

The preparation of cellulose nanocrystals from residue was conducted according to previously reported^10^ with slight modifications, which including delignification process and acid hydrolysis treatment. The residue was treated with the alkali solution (4% NaOH, w/v) and cooked at 165 °C for 90 min. Then the solid was washed with distilled water several times. Then, hydrogen peroxide (3%, v/v) and distilled water were added to ensure 12% concentration of pulp, followed by bleaching at 80 °C for 1 h. The mixture was filtered using excess distilled water. The acid hydrolysis treatment was conducted at 45 °C using concentrate sulphuric acid for 120 min under continuous stirring. The hydrolyzed material was washed by centrifugation at 10,000 rpm for 10 min. This step was repeated several times. And then the suspension was dialyzed using distilled water for several days until constant pH in the range of 5–6 was reached.

### Optical Microscope and Atomic Force Microscopy

A drop of the cellulose fiber suspension after bleaching was placed on a glass slide for imaging with an optical microscope (OLYMPUS BX51). A drop of CNC suspension was placed on a piece of freshly cleaved mica, dried and then analyzed with Atomic Force Microscope (AFM, Nanoscope IIIa Multimode 8, Bruker)^42^. Lengths and diameters were obtained using the section analysis tool of the NanoScope Analysis software (Bruker, version 1.40) from height mode AFM image. More than a hundred CNCs were randomly measured in order to determine the average length and diameter.

### Statistical Analysis

The data, expressed as the mean ± SD, included at least three replicates per sample. ANOVA and Tukey’s test were performed using Statistical Package for the Social Sciences (SPSS, version 21.0). Graphical representations were performed using Sigma plot version 12.3 (SPSS, Chicago, IL, USA). *P*-values < 0.05 were regarded as significant.

## Electronic supplementary material

supplement material
