# Supplementary material for: A full utilization of rice husk to evaluate phytochemical bioactivities and prepare cellulose nanocrystals
Source: Sci Rep. 2018 Jul 11;8:10482. doi: 10.1038/s41598-018-27635-3 (PMC6041302; doi:10.1038/s41598-018-27635-3)
Supplement: Supplementary file 1 — supplement material [file 41598_2018_27635_MOESM1_ESM.doc]

**Title:** **A** **full utilization of rice husk to evaluate phytochemical bioactivities and prepare cellulose nanocrystals**

**Authors: Yue Gao^1^, Xinbo Guo*, ^1^, Yu Liu****^2^, Zhiqiang Fang^2^, Mingwei Zhang^3^, Ruifen Zhang^3^, Lijun You^1^, Tong Li^4^, Rui Hai Liu*****^, 4^**

Supplement Figure-1. Chemical composition of the rice husk.
